# ﻿A new species of *Odorrana* Fei, Ye & Huang, 1990 (Amphibia, Anura, Ranidae) from central Guangxi, China with a discussion of the taxonomy of *Odorrana* (Bamburana)

**DOI:** 10.3897/zookeys.1190.109886

**Published:** 2024-01-25

**Authors:** Wei-Cai Chen, Yun-Ming Mo, Li Lin, Kun Qin

**Affiliations:** 1 Key Laboratory of Environment Change and Resources Use in Beibu Gulf Ministry of Education, Nanning Normal University, Nanning 530001, China; 2 Guangxi Key Laboratory of Earth Surface Processes and Intelligent Simulation, Nanning Normal University, Nanning 530001, China; 3 Natural History Museum of Guangxi, Nanning 530012, China; 4 Damingshan National Nature Reserve of Guangxi, Wuming 530114, China; 5 Guangxi Dayaoshan Forest Ecosystem Research Station, Jinxiu 545700, China

**Keywords:** Cryptic species, morphology, Odorous frog, phylogeny, taxonomy

## Abstract

A new species of odorous frog, *Odorranadamingshanensis***sp. nov.**, was found at the Damingshan National Nature Reserve in Guangxi, China. This species can be distinguished from its congeners by a combination of the following characters: medium body size (SVL 52.3–54.8 mm in males and 74.8–81.2 mm in females), sawtooth spinules on the upper lip, obtusely rounded snout that extends beyond the lower margin, distinct dorsolateral folds, horny tubercles on the rear of the back, presence of outer metatarsal tubercles, dilated nuptial pad with velvety spinules, distinct maxillary gland with tiny spines, and external lateral vocal sac. Through analysis of the 16S mitochondria gene, the new species is closely related to *O.nasica* and *O.yentuensis*, but the genetic divergence between the new species and the latter exceeds 7% (uncorrected *p*-distance). Currently, the new species is only known from its original discovery site. Furthermore, a discussion on the taxonomy of Odorrana (Bamburana) was conducted, identifying seven species within the subgenus Odorrana (Bamburana).

## ﻿Introduction

The genus *Odorrana* Fei, Ye & Huang, 1990 is currently known to consist of 62 species, primarily found in East and Southeast Asia (AmphibiaChina 2023; Frost 2023). Within China, there have been records of 40 species, with 18 species identified in Guangxi (AmphibiaChina 2023). Among these, *Odorranaversabilis* (Liu & Hu, 1962) was previously believed to have a wide distribution across southern and central China, including provinces such as Zhejiang, Guizhou, Anhui, Fujiang, Jiangxi, Hunan, Guangdong, Guangxi, and Hainan (Guo et al. 1966; Hu et al. 1973, 1978; Liu et al. 1973; Ma et al. 1982; Zou 1983; Pan et al. 1985; Zong and Ma 1985; Wu et al. 1986; Huang et al. 1990; Chen 1991; Sichuan Institute of Biology 1974; Fei et al. 2009; Mo et al. 2014; AmphibiaChina 2023).

Li et al. (2001) conducted a comparison of specimens from different geographic populations and observed distinct morphological divergences, indicating the presence of a species complex within *O.versabilis*. Based on both morphological and molecular data, Li et al. (2001) proposed that the *O.versabilis* species complex consisted of three separate species: *O.exiliversabilis* Li, Ye & Fei, 2001, *O.nasuta* Li, Ye & Fei, 2001, and *O.versabilis*. *Odorranaexiliversabilis* is found in Fujiang, Zhejiang, Anhui, and Jiangxi provinces, with Huangkeng County in Fujiang serving as the type locality (Li et al. 2001; AmphibiaChina 2023). *Odorrananasuta* is restricted to Hainan Island. *Odorranaversabilis*, on the other hand, occurs in Guizhou, Anhui, Jiangxi, Hunan, Guangdong, and Guangxi, with Longsheng and Jinxiu counties in Guangxi as the type locality. Subsequently, Fei et al. (2005) assigned these three species to the subgenus Bamburana within the genus *Odorrana*, based on several distinguishing characters. These included the presence of dorsolateral folds in the subgenus Bamburana (absent in the subgenus Odorrana), the upper lip adorned with sawtooth spinules (absent in the subgenus Odorrana), a xiphisternum without a notch (deeply notched in the subgenus Odorrana), and a widened posterior sternum (sternum not widened posteriorly in the subgenus Odorrana).

In 2010, Fei et al. conducted a revision of the genus *Odorrana*, reorganizing it into four separate and valid genera within the tribe Odorranini: *Bamburana*, *Eburana*, *Matsuirana*, and *Odorrana*. Fei et al. (2010) proposed that the genus *Bamburana* consisted of seven species: *B.exiliversabilis*, *B.montivaga* (Smith, 1921), *B.nasica* (Boulenger, 1903), *B.nasuta*, *B.trankieni* (Orlov, Le & Ho, 2003), *B.tormota* (Wu, 1977), and *B.versabilis*. However, the idea of dividing the tribe Odorranini into four genera did not gain widespread acceptance. In 2012, Fei et al. followed up on their previous classification (Fei et al. 2005), which had divided the genus *Odorrana* into the subgenera Odorrana (Odorrana) and Odorrana (Bamburana). Chen et al. (2013) subsequently confirmed the monophyly of the genus *Odorrana* and divided it into seven distinct clades (clades A–G). Clade F included *O.exiliversabilis*, *O.nasica*, *O.nasuta*, *O.tormota*, and *O.versabilis*, and corresponded to the subgenus Odorrana (Bamburana). However, Chen et al. (2013) did not specifically address the validity of Odorrana (Bamburana) but only confirmed that this group forms a monophyletic cluster. Furthermore, there was a lack of molecular data available to support the inclusion of *O.trankieni* in the subgenus Odorrana (Bamburana). To date, no further research has discussed the validity of Odorrana (Bamburana) or its constituent species.

The distribution of *O.versabilis* in Guangxi has been previously discussed by Fei et al. (2005) and Mo et al. (2014). They argued that it was widely present in counties such as Wuming, Shangsi, Longshen, Jinxiu, and Ziyuan. Previous studies have also indicated the occurrence of *O.nasuta* and *O.nasica* in Guangxi (Zhang and Wen 2000; Fei et al. 2005; Chen 2018; Huang et al. 2020). However, *O.nasuta* was specifically documented in Shiwandashan and Damingshan National Nature Reserves (Chen 2018; Huang et al. 2020), while information on the distribution of *O.nasica* is only available on AmphibiaChina (2023) without specifying its precise location. It is important to note that these findings are solely based on morphological descriptions and lack molecular evidence.

**Figure 1.**
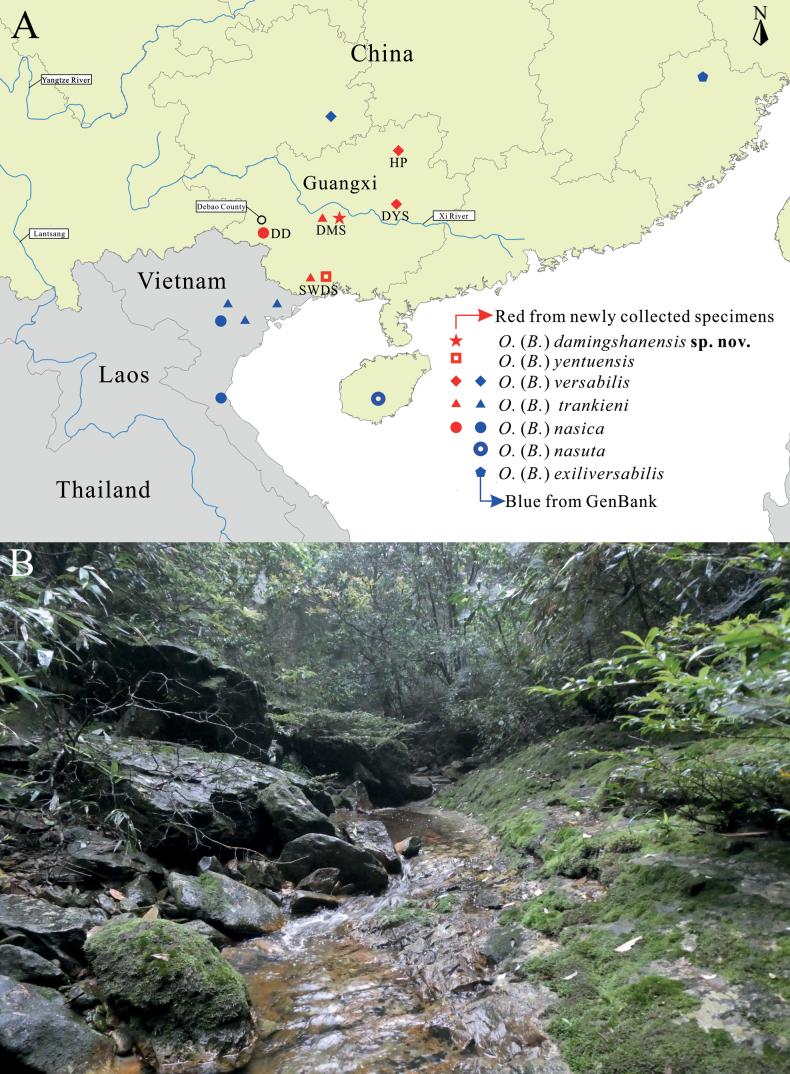
**A** distributions of the subgenus Odorrana (Bamburana), and **B** habitat of O. (B.) damingshanensis sp. nov. Abbreviations: DMS = Damingshan, DYS = Dayaoshan, SWDS = Shiwandashan, HP = Huaping, DD = Diding.

In recent years, we conducted herpetological surveys in various nature reserves in Guangxi (Fig. 1), where we collected a series of specimens resembling *O.versabilis*. However, through phylogenetic analyses, we discovered that these specimens did not form a monophyletic group. Instead, they were distributed across five distinct branches, suggesting the presence of cryptic species within the *O.versabilis* species complex. The objective of this study is to investigate the species diversity within the *O.versabilis* species complex, describe the potential new species that have been identified, and assess the validity of the subgenus Odorrana (Bamburana) and its constituent species.

## ﻿Material and methods

Fifty-four specimens from five different species were collected from various national nature reserves in Guangxi, China, including Shiwandashan (**SWDS**), Dayaoshan (**DYS**), Damingshan (**DMS**), Huaping (**HP**), and Diding (**DD**) reserves, between 2013 and 2022 (Fig. 1, Appendix 1). The specimens were euthanized using isoflurane, then fixed in 10% formalin and stored in 75% ethanol. Muscle tissues were obtained from each specimen before formalin fixation and preserved in 100% ethanol for molecular analyses (Luo et al. 2021). Morphological measurements were taken to the nearest 0.1 mm using digital calipers, following the methods described by Fei et al. (2009) and Luo et al. (2021). Abbreviations of characters used in the paper are as follows:

**SVL** snout-vent length, distance from the tip of snout to the posterior margin of vent;

**HDL** head length, distance from the tip of snout to the rear of jaw;

**HDW** maximum head width, the greatest width between the left and right articulations of jaw;

**SNT** snout length, distance from the tip of snout to the anterior corner of eye;

**EN** eye-nostril distance, distance from the anterior of eye to nostril;

**EYE** eye diameter, horizontally from the anterior to posterior corner of eye;

**IN** internarial space, the shortest distance between the inner margins of nostrils;

**IOD** interorbital distance, the shortest distance between the anterior corners of orbits;

**TMP** tympanum diameter;

**TEY** tympanum-eye distance, from the anterior edge of tympanum to the posterior corner of eye;

**TIB** tibia length, distance from knee to tarsus;

**THL** thigh length, distance from vent to knee;

**PL** pes length, distance from the tip of the fourth toe to the base of the inner metatarsal tubercles;

**FLL** forelimb length, distance from elbow to the tip of the third finger;

**ML** manus length, distance from the tip of the third digit to the base of tubercle on prepollex;

**FD_3_** diameter of the third finger disc;

**TD_4_** diameter of the fourth toe disc.

Genomic DNA was extracted from muscle tissues using DNeasy tissue extraction kits (Qiagen). Three fragments of mitochondrial DNA (mtDNA) were amplified, targeting segments of the 12S (~ 750 bp) and 16S (~ 1000 bp) ribosomal RNA genes, as well as the COI (~ 630 bp) gene. The primer sequences and PCR conditions followed the protocols outlined by Chen et al. (2013) for the 12S and 16S regions, and by Che et al. (2012) for the COI region. To confirm successful amplification, the PCR products were directly sequenced using an ABI 3730 automated DNA sequencer. The obtained sequences were then validated for accuracy and specificity through BLAST searches (Altschul et al. 1997) and deposited in GenBank (Table 1).

**Table 1.** Localities, voucher information and GenBank accession numbers for all samples used in this study.

ID	Species	Locality	Voucher	GenBank accession no.		
				12S	16S	COI
1	O. (B.) damingshanensis sp. nov.	Wuming, Guangxi, China	NNU00689	ON791444	ON791419	ON791392
2	O. (B.) damingshanensis sp. nov.	Wuming, Guangxi, China	NNU00690	ON791445	ON791420	ON791393
3	O. (B.) damingshanensis sp. nov.	Wuming, Guangxi, China	NNU00691	ON791446	ON791421	ON791394
4	O. (B.) damingshanensis sp. nov.	Wuming, Guangxi, China	NNU00692	ON791447	ON791422	ON791395
5	O. (B.) damingshanensis sp. nov.	Wuming, Guangxi, China	NNU00693	ON791448	ON791423	ON791396
6	O. (B.) nasica	Jingxi, Guangxi, China	NNU00663	ON791466	ON791443	ON791418
7	O. (B.) trankieni	Shangsi, Guangxi, China	NHMG1303003	ON791449	ON791424	
8	O. (B.) trankieni	Shangsi, Guangxi, China	NHMG140108	ON791451	ON791426	
9	O. (B.) trankieni	Shangsi, Guangxi, China	NHMG141103	MH665665	MH665671	ON791406
10	O. (B.) trankieni	Shangsi, Guangxi, China	NHMG141104	MH665666	MH665672	ON791405
11	O. (B.) trankieni	Shangsi, Guangxi, China	NHMG141107	MH665667	MH665673	ON791403
12	O. (B.) trankieni	Shangsi, Guangxi, China	NHMG141111	ON791450	ON791425	
13	O. (B.) trankieni	Shangsi, Guangxi, China	NHMG141113	MH665668	MH665674	ON791404
14	O. (B.) trankieni	Wuming, Guangxi, China	NNU20042913	ON791452	ON791427	ON791397
15	O. (B.) trankieni	Wuming, Guangxi, China	NNU20042914	ON791453	ON791428	ON791398
16	O. (B.) trankieni	Wuming, Guangxi, China	NNU20042915	ON791454	ON791429	ON791399
17	O. (B.) trankieni	Wuming, Guangxi, China	NNU20210302	ON791455	ON791430	ON791400
18	O. (B.) trankieni	Wuming, Guangxi, China	NNU20210303	ON791456	ON791431	ON791401
19	O. (B.) trankieni	Wuming, Guangxi, China	NNU20210304	ON791457	ON791432	ON791402
20	O. (B.) versabilis	Jinxiu, Guangxi, China	NNU00637	ON791460	ON791435	ON791409
21	O. (B.) versabilis	Jinxiu, Guangxi, China	NNU00638	ON791461	ON791436	ON791410
22	O. (B.) versabilis	Jinxiu, Guangxi, China	NNU00639	ON791462	ON791437	ON791411
23	O. (B.) versabilis	Jinxiu, Guangxi, China	NNU00640	ON791463	ON791438	ON791412
24	O. (B.) versabilis	Jinxiu, Guangxi, China	NNU00641	ON791464	ON791439	ON791413
27	O. (B.) versabilis	Jinxiu, Guangxi, China	NNU00647	ON791465	ON791440	ON791414
28	O. (B.) versabilis	Longsheng, Guangxi, China	NNU201908005	ON791458	ON791434	ON791407
29	O. (B.) versabilis	Longsheng, Guangxi, China	NNU201908010	ON791459	ON791433	ON791408
30	O. (B.) yentuensis	Shangsi, Guangxi, China	NHMG1401035	MH665669	MH665675	ON791416
31	O. (B.) yentuensis	Shangsi, Guangxi, China	NHMG1401036	ON791467	ON791441	ON791415
32	O. (B.) yentuensis	Shangsi, Guangxi, China	NNU00230	ON791468	ON791442	ON791417
33	*O.andersonii*	Longchuan, Yunnan, China	HNNU001YN topotype	KF185021	KF185057	
34	*O.anlungensis*	Anlong, Guizhou, China	HNNU1008I109 topotype	KF185013	KF185049	
35	*O.chapaensis*	Lai Chau, Vietnam	Genbank	DQ283372	DQ283372	
36	*O.chloronota*	Ha Giang, Vietnam	Genbank	DQ283394	DQ283394	
37	O. (B.) exiliversabilis	Wuyishan, Fujian, China	HNNU0607032 topotype	KF185020	KF185056	
38	O. (B.) exiliversabilis	Wuyishan, Fujian, China	LSU20200716WY02 topotype	MT934403	MT934403	MT934403
39	*O.fengkaiensis*	Fengkai C*O.*, Guangdong, China	SYS a002262 Paratype	KT315354	KT315375	
40	*O.grahami*	Kunming, Yunnan, China	HNNU1008II016 topotype	KF185015	KF185051	
41	*O.graminea*	Wuzhishan, Hainan, China	HNNU0606123 topotype	KF185002	KF185038	
42	*O.hainanensis*	Wuzhishan, Hainan, China	HNNU0606105 topotype	KF184996	KF185032	
43	*O.hejiangensis*	Hejiang, Sichuan, China	HNNU1007I202 topotype	KF185016	KF185052	
44	*O.hosii*	Kuala Lumpur, Malaysia	Genbank	AB511284	AB511284	
45	*O.huanggangensis*	Wuyishan, Fujian, China	HNNU0607001 paratype	KF185023	KF185059	
46	*O.ishikawae*	Amami Island, Ryukyu	Genbank	AB511282	AB511282	
47	*O.jingdongensis*	Jingdong, Yunan, China	20070711017 topotype	KF185014	KF185050	
48	*O.junlianensis*	Junlian, Sichuan, China	HNNU002 JL topotype	KF185022	KF185058	
49	*O.kuangwuensis*	Nanjiang, Sichuan, China	HNNU 0908II185 topotype	KF184998	KF185034	
50	*O.leporipes*	Shaoguan, Guangdong, China	HNNU1008I099 topotype	KF185000	KF185036	
51	*O.liboensis*	Maolan National Nature Reserve, Libo County, Guizhou, China	GZNU20180608007 holotype	MW481339	MW481350	
52	*O.lipuensis*	Lipu, Guangxi, China	NHMG1303018 paratype	MH665670	MH665676	
53	*O.lungshengensis*	Longsheng, Guangxi, China	HNNU70028 topotype	KF185018	KF185054	
54	*O.margaretae*	Dujiangyan City, Sichuan, China	SYS a003214	KT315370	KT315391	
55	*O.mutschmanni*	Cao Bang Province, Vietnam	IEBR 3723 holotype	KU356761	KU356765	
56	*O.nanjiangensis*	Nanjiang, Sichuan	HNNU1007I291 topotype	KF185006	KF185042	
57	*O.narina*	Okinawa Island, Ryukyu	Genbank	AB511287	AB511287	
58	O. (B.) nasica	Ha Tinh, Vietnam	AMNH A161169	DQ283345	DQ283345	
59	O. (B.) nasica	Tam Dao, Vinh Phu Prov., Vietnam	ROM 18031		DQ204493	
60	O. (B.) nasica	Tam Dao, Vinh Phu Prov., Vietnam	ROM 20235		DQ204494	
61	O. (B.) nasuta	Wuzhishan, Hainan, China	HNNU051119 topotype	KF185017	KF185053	
62	O. (B.) nasuta	Limu shan, Hainan, China	HNNU-A0019L	KX269223	KX269223	
63	*O.schmackeri*	Yichang, Hubei, China	HNNU 0908II349 topotype	KF185011	KF185047	
64	*O.swinhoana*	Taibei, Taiwan, China	HNNUTW1	KF185009	KF185045	
65	*O.tianmuii*	Linan, Zhejiang, China	HNNU 0707071 paratype	KF185004	KF185040	
66	*O.tiannanensis*	Hekou, Yunnan, China	HNNUHK001 topotype	KF185008	KF185044	
67	*O.tormota*	Huangshan, Anhui, China	AM04005, topotype	DQ835616	DQ835616	DQ835616
68	*O.tormota*	Huangshan, Anhui, China	SCUM052069	NC009423	NC009423	NC009423
69	O. (B.) trankieni	Son La province, Vietnam	VNMN04035	-	KX893900	
70	O. (B.) trankieni	Hoa Binh Province, Vietnam	IEBR A.2015.69	-	KX893889	
71	O. (B.) trankieni	Bac Giang Province, Vietnam	IEBR A.2013.74	-	KX893890	
72	O. (B.) versabilis	Leishan, Guizhou, China	HNNU003 LS	KF185019	KF185055	
73	*O.wuchuanensis*	Wuchuan, Guizhou, China	HNNU019 L topotype	KF185007	KF185043	
74	*O.yizhangensis*	Yizhang, Hunan, China	HNNU1008I075 topotype	KF185012	KF185048	
75	O. (B.) yentuensis	Vietnam	IEBR A.2015.38		KX893891	
76	*Babinadaunchina*	Emeishan, Sichuan, China	HNNU20060103 topotype	KF185029	KF185065	
77	*Ranachensinensis*	Ningshan, Shanxi, China	HNNU 20060359	KF185025	KF185061	

The DNA sequences obtained were aligned using the ClustalW algorithm implemented in Mega v. 7 (Kumar et al. 2016) with default settings. Homologous DNA sequences from GenBank were downloaded for phylogenetic analyses (Table 1). The uncorrected paired divergence (*p*-distance) was calculated using Mega v. 7. The best-fitting models of DNA substitution for the molecular data were determined using the Akaike Information Criterion (AIC) implemented in MrModeltest v. 2.3 (Nylander 2004), resulting in the selection of the GTR + I + G model. Phylogenetic relationships within the genus *Odorrana* were reconstructed using Bayesian inference (BI) with MrBayes v. 3.2 (Ronquist et al. 2012). A majority-rule consensus tree was constructed to calculate the Bayesian posterior probabilities (BPP) for the nodes in the tree. Maximum likelihood (ML) trees were inferred using the CIPRES Science Gateway server (https://www.phylo.org/portal2; Miller et al. 2010) with the estimation of the proportion of invariable sites and 1000 bootstrap pseudo replicates.

## ﻿Results

### ﻿Molecular analyses

**Figure 2.**
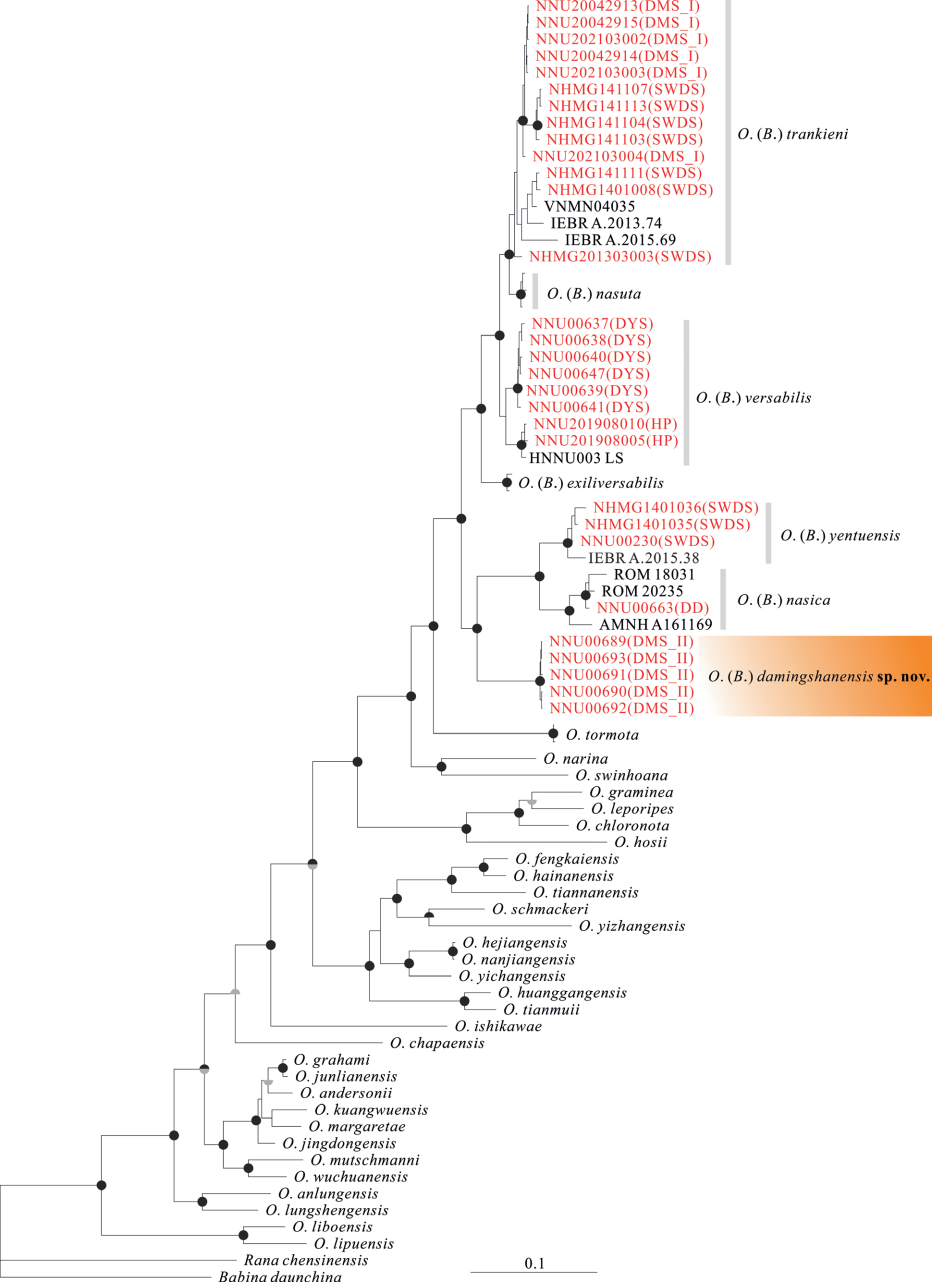
Maximum likelihood tree reconstructed based on 12S, 16S, and COI genes sequences. Note supports are shown on branches as bootstrap supports (upper half; > 70% < 90% = grey, > 90% = black) and Bayesian posterior probabilities (lower half; > 0.95 = grey, 1 = black). Red indicates the newly collected specimens in this study.

Both BI and ML analyses produced similar results, which align with previous studies conducted by Chen et al. (2013) and Luo et al. (2021). Our specimens were categorized into five distinct lineages based on preliminary phylogenetic analyses (Fig. 2). Firstly, the specimens from DMS were divided into two separate lineages. One lineage, referred to as DMS_I specimens, showed close relation to specimens from SWDS and *O.trankieni* from Vietnam, forming a monophyletic group. The other lineage, referred to as DMS_II specimens, formed a well-supported monophyletic group (BS = 100; PP = 1.00) and exhibited close affinity to *O.nasica* and *O.yentuensis*. The SWDS specimens, DMS_I specimens, and *O.trankieni* clustered together, forming a monophyletic group. The specimens from DYS and HP (type locality: Longsheng and Jinxiu counties, Guangxi, China), with *O.versabilis* from Leishan, Guizhou, formed a monophyletic group. The specimen from DD was found to be nested within *O.nasica* from Vietnam, indicating that they belong to the same species. *Odorranayentuensis* from SWDS and Vietnam formed a monophyletic group and showed close affinity to *O.nasica*. Lastly, *O.nasuta* from Hainan Island formed a distinct monophyletic group (Fig. 2).

The uncorrected *p*-distances for the 16S fragments within *O.versabilis* species complex are remarkably low, ranging from 1.3% to 3.1%. For instance, the genetic distances between *O.versabilis* and *O.nasuta* range from 1.8% to 2.4%, while those between *O.versabilis* and *O.nasica* range from 0.7% to 2.8% (Suppl. material 1: table S1). However, the DMS_II specimens show significant genetic divergences (> 5.0%) from their congeners. When examining the COI fragments, the smallest genetic distance among them is greater than 4.0% (Suppl. material 1: table S2). Specifically, the genetic distances between the specimens from DYS and SWDS range from 5.1% to 6.3%. On the other hand, the genetic divergences between the specimens from DMS and SWDS are very low, ranging from 0.3% to 0.8%, suggesting that these specimens are congeners. Nevertheless, the proposed new species (DMS_II specimens) exhibits distinct genetic differences from other similar species, with divergence values exceeding 5.0% for 16S and 10.0% for COI (Suppl. material 1: tables S1, S2).

### ﻿Morphological analyses

Table 2 presents the morphological differences observed. The DMS_II specimens can be distinguished from other similar species by various morphological characteristics listed in Table 2, such as SVL, vocal sac, horny tubercles on the rear of the back, and sawtooth spinules on the upper lip. For further information, please refer to the Taxonomic account section below.

**Table 2.** Morphological comparisons. Abbreviations: DMS = Damingshan, DYS = Dayaoshan, SWDS = Shiwandashan, SVL = snout-vent length, SD = Standard deviation.

Character	SVL (mean ± SD, ranges)		SVL of female / SVL of male	Tip of snout strongly projecting beyond margin of the lower jaw	Vocal sac	Rear of the back with horny tubercles	Pineal ocellus	Sternum widened posteriorly without notch	Upper lip with sawtooth spinules	Relative lengths of fingers	References
	Male	Female									
DMS_II O. (B.) damingshanensis sp. nov.	53.3 ± 1.3 (52.3–54.8) *n* = 3	78.0 ± 4.5 (74.8–81.2) *n* = 2	1.46	No	External	Yes	Yes	Yes	Yes	II < IV < I < III	This study
DYS O. (B.) versabilis	76.4 ± 4.1 (71.7–79.4) *n* = 3	78.0 ± 1.9 (77.0–82.4) *n* = 7	1.02	Yes	Internal	No	Yes	Yes	Yes	II < IV < I < III	This study
DMS_I O. (B.) trankieni	72.5 ± 2.9 (70.2–76.2) *n* = 4	75.4 ± 5.9 (69.0–80.6) *n* = 3	1.04	Yes	External	No	Yes	Yes	Yes	IV < II < I < III	This study
SWDS O. (B.) trankieni	71.7 ± 1.3 (70.0–73.1) *n* = 5	80.4 ± 3.5 (76.2–83.9) *n* = 4	1.12	Yes	External	No	Yes	Yes	Yes	IV < II < I < III	This study
SWDS O. (B.) yentuensis	43.7 ± 1.3 (40.1–46.9) *n* = 13	59.9 ± 1.3 (54.1–65.3) *n* = 6	1.37	No	External	Yes	Yes	Yes	Yes	II < I < IV < III	This study
O. (B.) exiliversabilis	48.7 (42.7–52.4) *n* = 20	58.1 (51.8–61.8) *n* = 24	1.19	No	Internal	No	Yes	Yes	Yes	II < IV < I < III	Fei et al. 2001, 2012; Li et al. 2001; AmphibiaChina 2023
O. (B.) nasica	41.0–46.0; *n* = 4	60.0–70.7; *n* = 2	/	Yes	External	No	No	Yes	Yes	II < IV < I < III	Boulenger 1903; Zhang and Wen 2000; Stuart and Chan-ard 2005; Tran et al. 2008; Yang 1991
O. (B.) nasuta	59.2 (57.1–63.2) *n* = 10	73.4 (73.1–73.6) *n* = 2	1.24	Yes	External	No	Yes	Yes	Yes	II < IV < I < III	Fei et al. 2001, 2012; Li et al. 2001; Chen 2018; Huang et al. 2020; AmphibiaChina 2023
*O.tormota*	33.8 (32.0–36.3)	59.5 (59.0–60.0)	1.76	No	External	No	–	No	No	I ≈ II < IV < III	Wu 1977; Fei et al. 2012; AmphibiaChina 2023
O. (B.) trankieni	75.2–84.1, *n* = 7	86.2–95.8, *n* = 5	/	Yes	External	No	–	Yes	Yes	IV < II < I < III	Orlov et al. 2003; Pham et al. 2020
O. (B.) versabilis	72.4 (69.3–77.8) *n* = 5	77.0 (70.0–81.4) *n* = 6	1.06	Yes	Internal	No	Yes	Yes	Yes	II < IV < I < III	Liu and Hu 1962; Fei et al. 2001, 2012; Li et al. 2001; AmphibiaChina 2023
O. (B.) yentuensis	44.5 (44.3–45.5) *n* = 4	60.9 (59.3–61.9) *n* = 6	1.37	No	External	Yes	Yes	Yes	Yes	II < I < IV < III	Tran et al. 2008; Lu et al. 2016

### ﻿Taxonomic results

Based on the analysis of morphological characters and molecular data, it has been determined that the specimens from SWDS, DMS_I, and those previously identified as *O.trankieni* from Vietnam, belong to the same species, namely *O.trankieni*. The specimens from HP and DYS have been identified as *O.versabilis*. The DD specimen has been classified as *O.nasica*. Furthermore, the DMS_II specimens have been found to represent an undescribed species of *Odorrana*, which will be described below.

#### ﻿ Odorrana damingshanensis sp. nov.

Taxon classificationAnimaliaAnuraRanidae

34908253-4A07-518E-8547-9CC694376481

https://zoobank.org/448AD82C-76AE-4D51-88DF-D7790C2FC408

Figs 3 , 4

##### Type material examined.

***Holotype*.**NNU 00690, adult male, from the Damingshan National Nature Reserve, Wuming District, Nanning City, Guangxi, China (23.4637°N, 108.4869°E; elevation 1159 m), collected by Weicai Chen on 25 April 2022. ***Paratypes*.**NNU 00689 and NNU 00691, adult males; NNU 00692 and NNU 00693, adult females, collected at the same site and time as the holotype.

##### Diagnosis.

Based on both molecular analyses and specific morphological traits, these specimens were assigned to the genus *Odorrana*. The distinguishing morphological characteristics of these species include dilated and tapering tips of the digits, disks with circummarginal grooves and a longer vertical diameter than horizontal diameter, fully webbed toes, the absence of a tarsal fold, a thick first finger with a distinct nuptial pad, sawtooth spinules on the upper lip, and well-defined dorsolateral folds (Fei et al. 2001, 2005).

**Figure 3.**
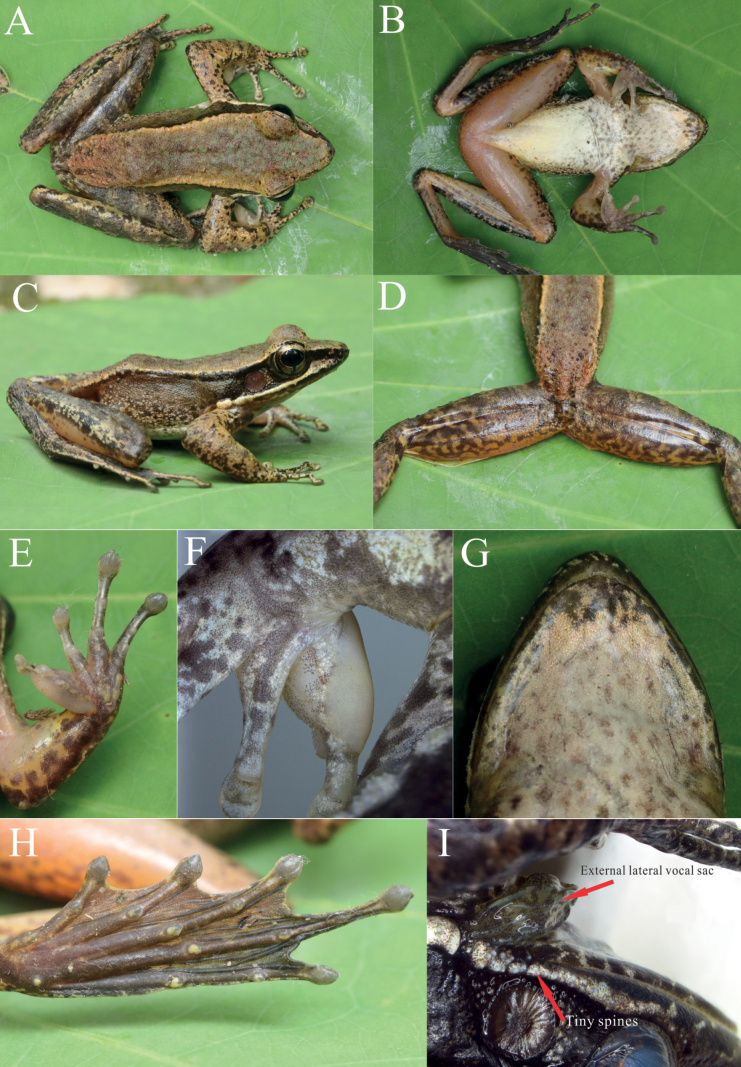
The holotype of O. (B.) damingshanensis sp. nov. (NNU 00690) **A** dorsal view **B** ventral view **C** dorsolateral view **D** rear of the back with horny tubercles and dorsal view of thighs **E** ventral view of hand **F** nuptial pad with velvety spinules **G** ventral view of snout **H** ventral view of foot, and **I** external lateral vocal sac and tiny spines on maxillary glands.

*Odorranadamingshanensis* sp. nov. can be distinguished from other species in its genus by the following combination of characters: (1) medium body size (SVL 52.3–54.8 mm in males and 74.8–81.2 mm in females); (2) SVL of female/SVL of male = 1.46; (3) sawtooth spinules on the upper lip; (4) snout obtusely rounded without significantly extending beyond the lower jaw; (5) well-defined dorsolateral folds; (6) horny tubercles on the rear of the back; (7) presence of outer metatarsal tubercles; (8) absence of a supratympanic fold; (9) highly dilated nuptial pad with velvety spinules on the dorsolateral surface of Finger I; (10) distinct expansion of the tips of the second, third, and fourth fingers, with the first slightly enlarged; expanded tips of the toes with distinct circummarginal grooves; (11) distinct maxillary gland with tiny spines; and (12) external lateral vocal sac (Fig. 3A–I).

##### Description of holotype.

Head longer than wide (HDL/HDW = 1.23); snout obtusely rounded in dorsal view, but not strongly projecting beyond margin of lower jaw (Fig. 3G); canthus rostral distinct, loreal region concave; nostrils oval, oblique, and closer to tip of snout than eye; upper lip with sawtooth spinules (Fig. 3G); eye diameter less than snout length (EYE/SNT = 0.88); interorbital region flat with a pineal; interorbital distance less than eye diameter (IOD/EYE = 0.75); internostril distance less than eye diameter (IN/EYE = 0.88); tympanum distinct, rounded, 57% eye diameter, slightly concave relative to skin of temporal region; supratympanic fold absent; vomerine teeth on two oblique ridges, closed each other than choana; tongue elongated, deeply notched posteriorly; pupil horizontally oval; and external lateral vocal sac (Fig. 3I).

Forelimbs stout, relative length of fingers II < IV < I < III; tips of the second, third and fourth distinctly expanded, but the first slightly enlarged; tips of all fingers with circummarginal grooves; Finger III disk width less than tympanum diameter (FD_3_/TMP = 0.62); finger webbing absent; subarticular tubercles prominent, rounded, formula 1, 1, 2, 2; inner and outer palmar tubercles distinct; nuptial pad on lateral surface of Finger I strongly dilated with velvety spinules, extending from hand base to level of subarticular tubercle (Fig. 3E, F). Tips of toes expanded, with distinct circummarginal grooves; relative length of toes I < II < III ≈ V < IV; toes entirely webbed; subarticular tubercles distinct, rounded, formula 1, 1, 2, 3, 2; inner metatarsal tubercle elongated; outer metatarsal tubercle conical (Fig. 3H).

Body surface shagreened; rear of the back with horny tubercles; ventral surface of venter, forelimbs, and thighs smooth; flanks shagreened; dorsal of forelimbs and hindlimbs shagreened, and hindlimbs with sparse tubercles; two distinct maxillary glands with tiny spinules (Fig. 3I).

##### Coloration in life.

Dorsum grey-beige, with irregular grass-green blotches; a discrete darker brown stripe from tip of snout, across canthus rostral, along the inferior dorsolateral fold, finally ending at the anterior of groin; pineal gland grass-green; cream white stripe from anterior of upper lip to maxillary glands; tympanic region brown, some creamy white tubercles around the tympanum; the upper part of belly, chest, and throat with irregular grey cloud, but the lower part of belly creamy white without spots; ventral surface of thighs and forelimbs incarnadine without spots; forelimbs and hindlimbs with pale brown crossbars, three on lower arm, four on thigh and four on tibia; pupil black with orange border; iris creamy yellow, but the posterior iris pale jacinth; and velvety nuptial pad creamy white (Fig. 3A–I).

##### Coloration in preservative.

Dorsum brown; bars on forelimbs and hindlimbs darker brown; horny tubercles on the rear of the back turned into creamy white; creamy white nuptial pad turned into grey; the upper part of belly, throat, and chest with brown cloud; the lower part of belly immaculate creamy white; the ventral surface of the hindlimbs creamy yellow; external lateral vocal sac pale green and projecting distinctly (Fig. 3I).

##### Etymology.

The specific name of this species, *damingshanensis*, is derived from its discovery locality, Damingshan National Nature Reserve. In English, it is suggested to be called the Damingshan Bamboo-leaf Frog. In Chinese, it is known as大明山竹叶蛙(Dà Míng Shān Zhú Yè Wā).

##### Distribution and ecology.

**Figure 4.**
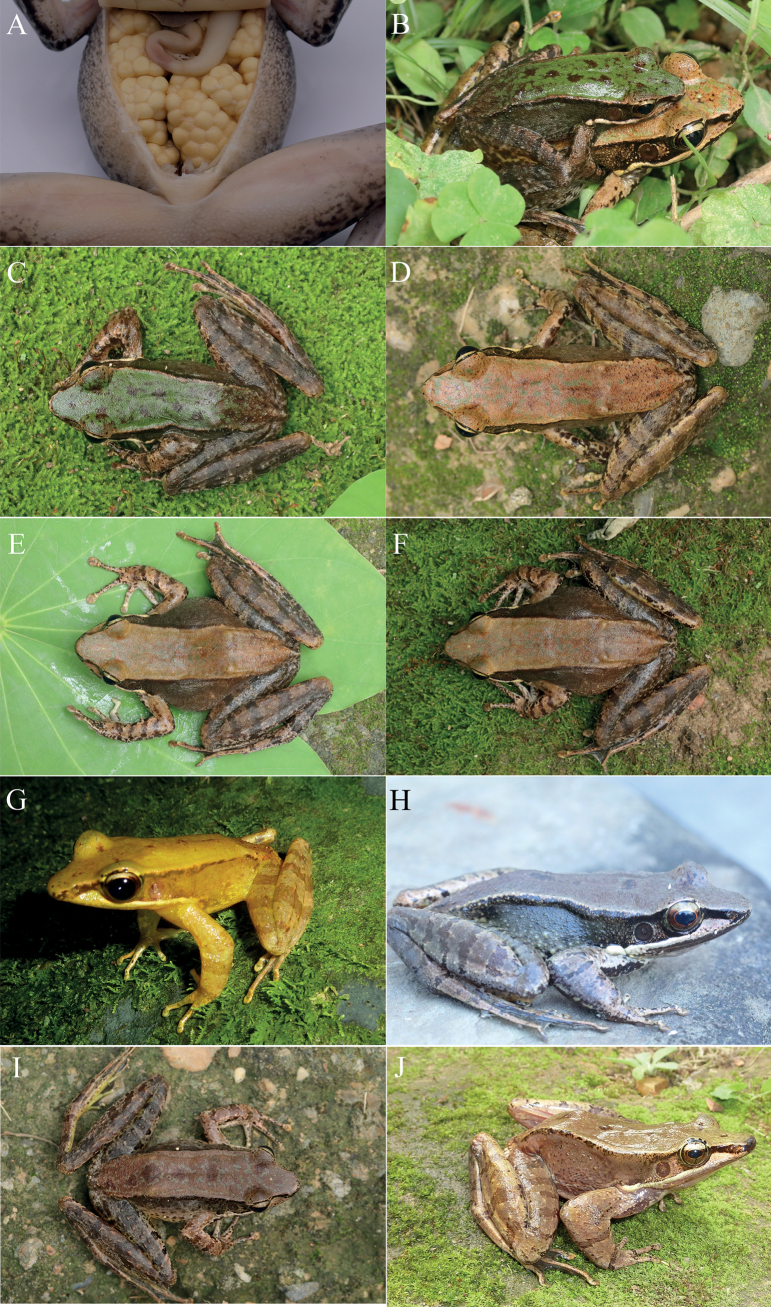
**A** female with creamy yellow eggs without pigmented poles **B** amplexus **C** dorsal view of NNU 00691 **D** dorsal view of NNU 00689 **E** dorsal view of NNU 00692 **F** dorsal view of NNU 00693 **G** dorsolateral view of O. (B.) yentuensis (NHMG1401036, adult male) **H** dorsolateral view of O. (B.) versabilis (NNU00638, adult male) **I** dorsal view of O. (B.) nasica (NNU00663, adult female) **J** dorsolateral view of O. (B.) trankieni (NHMG141107, adult male).

*Odorranadamingshanensis* sp. nov. is a newly discovered species found in the Damingshan National Nature Reserve, located in Wuming District, Nanning City, Guangxi, China. This species was observed near slow-flowing rocky streams, which were ~ 2–3 m wide and 10–30 cm deep (Fig. 1B). The frogs were often seen sitting on rocks, and interestingly, one specimen (NNU 00691) was found on a leaf near a stream, while another specimen (NNU 00693) was perched on a dry branch above a stream. The surrounding vegetation in the area consists of evergreen forest, and the elevation is ~ 1200 m with an ambient temperature of 20 °C in April. During the survey, no advertisement calls were heard; however, both female specimens were gravid, carrying creamy yellow eggs without black poles (Fig. 4A). Additionally, amplexus behavior was observed when males encountered females indoors (Fig. 4B). The breeding season of *O.damingshanensis* sp. nov. is speculated to occur between April and May. Other sympatric species in the area include *Gracixalusjinxiuensis* and *Quasipaashini*.

##### Sexual dimorphism and variation.

The measurements of *O.damingshanensis* sp. nov. are provided in Table 3. Females of this species were observed to be significantly larger than males in terms of SVL, with a ratio of 1.46. The specimens show variation in dorsal colors: NNU 00691 displays a grass green coloration with brown blotches (Fig. 4C), while NNU 00689 exhibits a pale beige coloration with grass green blotches (Fig. 4D). Males have a higher density of horny tubercles on the rear of their backs compared to females (Fig. 4C–F).

**Table 3.** Measurements of O. (B.) damingshanensis sp. nov. (in mm). Abbreviations are defined in the text.

Characters	NNU 00689	NNU 00690	NNU 00691	NNU 00692	NNU 00693
Sex	Male	Male	Male	Female	Female
SVL	52.3	54.8	52.8	74.8	81.2
HDL	19.2	20.4	18.0	25.5	27.4
HDW	16.0	16.6	16.6	23.1	25.9
SNT	7.7	7.8	7.7	11.1	11.6
EN	4.0	4.3	4.2	5.2	5.7
EYE	6.4	6.9	6.6	8.8	8.3
IN	5.7	6.1	6.2	8.4	8.1
IOD	4.9	5.2	5.2	7.5	7.4
TMP	3.7	3.9	3.8	5.0	4.7
TEY	1.9	2.1	1.7	2.8	3.0
TIB	30.3	32.1	32.5	44.2	48.9
THL	27.0	29.3	28.2	39.4	44.0
PL	29.5	29.3	28.7	41.4	43.7
FLL	23.0	25.4	25.2	35.7	38.6
ML	13.6	14.6	14.0	21.1	23.5
FD_3_	2.1	2.4	2.2	3.1	3.4
TD_4_	1.8	2.0	1.8	2.6	2.8

##### Comparisons.

*Odorranadamingshanensis* sp. nov. shares morphological similarities with closely phylogenetically related species, including *O.exiliversabilis*, *O.nasica*, *O.nasuta*, *O.tormota*, *O.trankieni*, *O.versabilis*, and *O.yentuensis* (Fig. 4G–J, Table 2). These similarities can be observed in its protruding snout, slender limbs, sawtooth spinules on the upper lip, and distinct dorsolateral folds. However, *O.damingshanensis* sp. nov. can be distinguished from *O.exiliversabilis* by the noticeably larger body size in females (SVL 74.8–81.2 mm vs SVL 51.8–61.8 mm); presence of horny tubercles on the rear of the back (vs absence); and presence of an external lateral vocal sac (vs an internal subgular vocal sac) (Fei et al. 2001, 2012; Li et al. 2001). *Odorranadamingshanensis* sp. nov. differs from *O.nasica* in having a relatively larger body size in males (SVL 52.3–54.8 mm vs SVL 41.0–46.0 mm); absence of strongly protruding snout (vs presence); a distinct pineal body (vs invisible); an elongated inner metatarsal tubercle (vs an oval inner metatarsal tubercle); absence of whitish spinules scattered ventrally near the groin (vs presence of such spinules) (Yang 1991; Zhang and Wen 2000; Stuart and Chan-ard 2005; Tran et al. 2008). *Odorranadamingshanensis* sp. nov. differs from *O.nasuta* in having a relatively smaller body size in males (SVL 52.3–54.8 mm vs SVL 57.1–63.2 mm); SVL of female/SVL of male = 1.46 (vs the ratio of 1.24), distinct maxillary gland with tiny spines (vs absent tiny spines on maxillary gland); presence of horny tubercles on the rear of the back (vs smooth); absence of strongly protruding snout (vs presence) (Fei et al. 2001, 2012; Li et al. 2001). *Odorranadamingshanensis* sp. nov. differs from *O.tormota* by the absence of a deeply sunk tympanum forming an external auditory canal (vs presence of a deeply sunk tympanum); conspicuously larger body size (SVL 52.3–54.8 mm in males and 74.8–81.2 mm in females vs SVL 32.0–36.3 mm in males and 59.30–60.0 mm in females); presence of sawtooth spinules on upper lip (vs absence) (Wu 1977; Fei et al. 2012; AmphibiaChina 2023). *Odorranadamingshanensis* sp. nov. differs from *O.trankieni* in having a conspicuously smaller body size (SVL 52.3–54.8 mm in males and 74.8–81.2 mm in females vs SVL 75.2–84.1 mm in males and 86.8–95.9 mm in females); SVL of female/SVL of male = 1.46 (vs similar body sizes for males and females); absence of strongly protruding snout (vs presence); presence of horny tubercles on the rear of the back (vs smooth); relative lengths of fingers II < IV < I < III (vs IV < II < I < III); distinct maxillary gland with tiny spines (vs absent tiny spines)(Orlov et al. 2003; Pham et al. 2020). *Odorranadamingshanensis* sp. nov. differs from *O.versabilis* in having a conspicuously smaller body size in males (SVL 52.3–54.8 mm vs SVL 70.4–77.2 mm); presence of horny tubercles on the rear of the back (vs smooth); distinct maxillary gland with tiny spines (vs absent tiny spines); external lateral vocal sac (vs internal subgular vocal sac) (Liu and Hu 1962; Fei et al. 2001, 2012; Li et al. 2001). *Odorranadamingshanensis* sp. nov. differs from *O.yentuensis* in having a notably larger body size (SVL 52.3–54.8 mm in males and 74.8–81.2 mm in females vs SVL 41.7–46.2 mm in males and 59.3–65.7 mm in females); shagreened body surface (vs smooth); distinct maxillary gland with tiny spines (vs absent tiny spines on maxillary gland); irregular grey cloud on the upper part of belly, chest, and throat, but creamy white the lower part of belly without spots (vs yellowish white ventral side of body without spots)(Tran et al. 2008; Lu et al. 2016).

Finally, *O.damingshanensis* sp. nov. can be distinguished from other *Odorrana* species by the presence of sawtooth spinules on the upper lip (vs absent sawtooth spinules on the upper lip, *O.absita*, *O.amamiensis*, *O.andersonii*, *O.anlungensis*, *O.arunachalensis*, *O.aureola*, *O.bacboensis*, *O.banaorum*, *O.bolavensis*, *O.cangyuanensis*, *O.chapaensis*, *O.chloronota*, *O.concelata*, *O.dulongensis*, *O.fengkaiensis*, *O.geminata*, *O.gigatympana*, *O.grahami*, *O.graminea*, *O.hainanensis*, *O.heatwolei*, *O.hejiangensis*, *O.hosii*, *O.huanggangensis*, *O.ichangensis*, *O.indeprensa*, *O.ishikawae*, *O.jingdongensis*, *O.junlianensis*, *O.khalam*, *O.kuangwuensis*, *O.kweichowensis*, *O.leporipes*, *O.liboensis*, *O.lipuensis*, *O.livida*, *O.lungshengensis*, *O.macrotympana*, *O.margaretae*, *O.mawphlangensis*, *O.monjerai*, *O.morafkai*, *O.mutschmanni*, *O.nanjiangensis*, *O.narina*, *O.orba*, *O.sangzhiensis*, *O.schmackeri*, *O.sinica*, *O.splendida*, *O.supranarina*, *O.swinhoana*, *O.tianmuii*, *O.tiannanensis*, *O.tormota*, *O.utsunomiyaorum*, *O.wuchuanensis*, and *O.yizhangensis*); the presence of external lateral vocal sac (vs absent vocal sac, *O.arunachalensis*, *O.concelata*, *O.heatwolei*, *O.hosii*, *O.ichangensis*, *O.kuangwuensis*, *O.leporipes*, *O.liboensis*, *O.lipuensis*, *O.livida*, *O.margaretae*, *O.mawphlangensis*, *O.monjerai*, *O.mutschmanni*, *O.narina*, *O.sangzhiensis*, *O.schmackeri*, *O.splendida*, *O.supranarina*, *O.wuchuanensis*); well-defined dorsolateral folds (vs absent dorsolateral folds, *O.andersonii*, *O.anlungensis*, *O.arunachalensis*, *O.aureola*, *O.bacboensis*, *O.cangyuanensis*, *O.chapaensis*, *O.chloronota*, *O.concelata*, *O.dulongensis*, *O.fengkaiensis*, *O.geminata*, *O.grahami*, *O.hainanensis*, *O.heatwolei*, *O.hejiangensis*, *O.huanggangensis*, *O.ichangensis*, *O.ishikawae*, *O.jingdongensis*, *O.junlianensis*, *O.kuangwuensis*, *O.kweichowensis*, *O.liboensis*, *O.lipuensis*, *O.lungshengensis*, *O.macrotympana*, *O.margaretae*, *O.mawphlangensis*, *O.morafkai*, *O.mutschmanni*, *O.nanjiangensis*, *O.sangzhiensis*, *O.schmackeri*, *O.sinica*, *O.splendida*, *O.swinhoana*, *O.tianmuii*, *O.tiannanensis*, *O.wuchuanensis*, and *O.yizhangensis*); horny tubercles on the rear of the back (vs absent horny tubercles on the rear of the back, *O.absita*, *O.amamiensis*, *O.andersonii*, *O.anlungensis*, *O.arunachalensis*, *O.aureola*, *O.bacboensis*, *O.banaorum*, *O.bolavensis*, *O.cangyuanensis*, *O.chapaensis*, *O.chloronota*, *O.concelata*, *O.dulongensis*, *O.fengkaiensis*, *O.geminata*, *O.gigatympana*, *O.grahami*, *O.graminea*, *O.hainanensis*, *O.heatwolei*, *O.hejiangensis*, *O.hosii*, *O.huanggangensis*, *O.ichangensis*, *O.indeprensa*, *O.ishikawae*, *O.jingdongensis*, *O.junlianensis*, *O.khalam*, *O.kuangwuensis*, *O.kweichowensis*, *O.leporipes*, *O.liboensis*, *O.lipuensis*, *O.livida*, *O.lungshengensis*, *O.macrotympana*, *O.margaretae*, *O.mawphlangensis*, *O.monjerai*, *O.morafkai*, *O.mutschmanni*, *O.nanjiangensis*, *O.narina*, *O.orba*, *O.sangzhiensis*, *O.schmackeri*, *O.sinica*, *O.splendida*, *O.supranarina*, *O.swinhoana*, *O.tianmuii*, *O.tiannanensis*, *O.utsunomiyaorum*, *O.wuchuanensis*, and *O.yizhangensis*).

## ﻿Discussion

The specimens in our study were classified into five species: *O.damingshanensis* sp. nov., *O.nasica*, *O.trankieni*, *O.versabilis*, and *O.yentuensis*, indicating a significant diversity of species in Guangxi, China. Our findings challenge previous research (Zhang and Wen 2000; Fei et al. 2005; Chen 2018; Huang et al. 2020) that identified SWDS and DMS_I specimens as *O.trankieni* instead of *O.nasuta* or *O.nasica*, based on morphology and phylogeny (Fig. 2). Chen (2018) and Huang et al. (2020) suggested the presence of *O.nasuta* in SWDS and DMS but lacked molecular data to support their claims. By combining phylogenetic and morphological data, we confirm that Chen (2018) and Huang et al. (2020) misidentified their specimens (Voucher nos.: NHMG 1303003, NHMG 141103–04 for Chen; Huang 201808296–98 for Huang et al.). Zhang and Wen (2000) proposed the occurrence of *O.nasica* in Debao County and DMS (voucher no. 830354, female, SVL 60.0 mm, collected in Debao County, Guangxi, China). However, they did not provide any supporting evidence for their findings. Upon examining the description provided by Zhang and Wen (2000), we observed that our DMS_I specimens exhibited similar body size, color pattern, and other diagnostic features to their specimen (Table 2; Zhang and Wen 2000). Considering the geographical proximity of Debao County to DD, we conclude that the Debao specimen and DD specimen represent the same species, *O.nasica*.

Upon examination of the series of specimens collected across Guangxi, we discovered that *O.damingshanensis* sp. nov., *O.nasica*, *O.nasuta*, *O.trankieni*, and *O.versabilis* exhibit similar body sizes and morphological characteristics in females, but not in males (Table 2). These factors have often led to misidentification of these species. Fig. 1 indicates that *O.exiliversabilis* and *O.versabilis* are present in the northern region of the Xi River, while *O.damingshanensis* sp. nov., *O.nasica*, *O.nasuta*, and *O.trankieni* are found in the southern region of the Xi River. However, further research is required to determine the extent to which the Xi River plays a significant role in the separation of these species. Currently, we know that *O.exiliversabilis* occurs in Southeastern China (Fujian, Zhejiang, Anhui, and Jiangxi provinces), *O.nasica* is found in the Sino-Vietnamese region (Yunnan, Guangxi, and Northern Vietnam), *O.nasuta* is limited to Hannan Island, *O.trankieni* is distributed in Northern Vietnam (Son La, Hoa Binh, and Bac Giang provinces) and Guangxi, China (this study), representing a new country record for China. *O.versabilis* inhabits Southern China (Guizhou, Guangxi, Guangdong, Hunan, and Jiangxi provinces), and *O.yentuensis* occurs in the Sino-Vietnamese region (AmphibiaChina 2023; Frost 2023). *Odorranadamingshanensis* sp. nov. is only known from DMS. It should be noted that *O.damingshanensis* sp. nov. and *O.trankieni* are sympatric species. However, *O.trankieni* is typically found at lower altitudes ranging from 200 to 900 m, while *O.damingshanensis* sp. nov. is found at higher altitudes exceeding 1000 m. *Odorranatrankieni* is commonly encountered in small cascade streams or wide streams with a slow current, whereas *O.damingshanensis* sp. nov. was specifically found in small, slow-flowing rocky streams (Fig. 1B).

Fei et al. (2005) initially proposed dividing the genus *Odorrana* into two subgenera, Odorrana (Bamburana) and Odorrana (Odorrana), based on several distinguishing characters. These characters included the presence of dorsolateral folds (absent in the latter), upper lip with sawtooth spinules (absent in the latter), xiphisternum without a notch (deeply notched in the latter), and posterior widening of the sternum (not widened in the latter). In a subsequent revision by Fei et al. (2010), the genus *Odorrana* was elevated to the generic level and divided into four genera within the tribe Odorranini: *Bamburana*, *Eburana*, *Matsuirana*, and *Odorrana*. Fei et al. (2010) suggested that the genus *Bamburana* comprised seven species: *B.exiliversabilis*, *B.montivaga*, *B.nasica*, *B.nasuta*, *B.tormota*, *B.trankieni*, and *B.versabilis*. However, this revision has not been widely accepted (AmphibiaChina 2023; Frost 2023). In a subsequent study by Fei et al. (2012), they did not adopt this revision and instead followed their original proposal of Odorrana (Bamburana) and Odorrana (Odorrana) (Fei et al. 2005). Phylogenetically, Chen et al. (2013) confirmed the monophyly of the genus *Odorrana* and divided it into seven major branches (clades A–G). They did not support the genera *Bamburana*, *Eburana*, *Matsuirana*, and *Odorrana* proposed by Fei et al. (2010): *Bamburana*, *Eburana*, and *Matsuirana* formed monophyletic groups, while *Odorrana* was paraphyletic. Furthermore, except for *Bamburana*, no diagnostic characters corresponded to the four genera proposed by Fei et al. (2010). According to Fei et al. (2005), the genus *Odorrana* can be divided into two subgenera: Odorrana (Bamburana) and Odorrana (Odorrana). By following this classification, differentiating Odorrana (Bamburana) from Odorrana (Odorrana) becomes easier based on four distinct characteristics: distinct dorsolateral folds, sawtooth spinules on the upper lip, absence of a notch in the xiphisternum, and posterior widening of the sternum. Our phylogenetic trees also provide support for the monophyly of Odorrana (Bamburana) species (BS = 100; PP = 1.00), which include eight species: O. (B.) damingshanensis sp. nov., O. (B.) exiliversabilis, O. (B.) nasica, O. (B.) nasuta, O. (B.) tormota, O. (B.) trankieni, O. (B.) versabilis, and O. (B.) yentuensis. However, it is important to note that O. (B.) tormota has a deeply sunk tympanum forming an external auditory canal (Wu 1977). Furthermore, O. (B.) tormota lacks sawtooth spinules on the upper lip and has a deep notch on the xiphisternum. Therefore, we suggest excluding O. (B.) tormota from the subgenus Odorrana (Bamburana). Further investigation is necessary to determine if the subgenus Odorrana (Odorrana) can be further subdivided.

In this study, we identified five of seven species of the subgenus Odorrana (Bamburana) that occur in Guangxi: O. (B.) damingshanensis sp. nov., O. (B.) nasica, O. (B.) trankieni, O. (B.) versabilis, and O. (B.) yentuensis. This finding highlights the significant species diversity of Odorrana (Bamburana) in Guangxi. It is worth noting that the presence of O. (B.) trankieni is a new record for China.

## Supplementary Material

XML Treatment for Odorrana damingshanensis

## ﻿Additional information

### Author ORCIDs

Wei-Cai Chen https://orcid.org/0000-0002-2398-4079

### Data availability

All of the data that support the findings of this study are available in the main text or Supplementary Information.

## ﻿Appendix 1

**Table A1.** Specimens examined. Abbreviations: NNU = Nanning Normal University; NHMG = Natural History Museum of Guangxi.

ID	Species	Vouchers	Sex	Locality
1	O. (B.) damingshanensis sp. nov.	NNU00689	Adult, male	Wuming, Guangxi, China
2	O. (B.) damingshanensis sp. nov.	NNU00690	Adult, male	Wuming, Guangxi, China
3	O. (B.) damingshanensis sp. nov.	NNU00691	Adult, male	Wuming, Guangxi, China
4	O. (B.) damingshanensis sp. nov.	NNU00692	Adult, female	Wuming, Guangxi, China
5	O. (B.) damingshanensis sp. nov.	NNU00693	Adult, female	Wuming, Guangxi, China
6	O. (B.) damingshanensis sp. nov.	NNU00201	Sub-adult	Wuming, Guangxi, China
7	O. (B.) nasica	NNU00663	Adult, female	Jingxi, Guangxi, China
8	O. (B.) trankieni	NHMG1303003	Adult, male	Shangsi, Guangxi, China
9	O. (B.) trankieni	NHMG140108	Adult, female	Shangsi, Guangxi, China
10	O. (B.) trankieni	NHMG141103	Adult, female	Shangsi, Guangxi, China
11	O. (B.) trankieni	NHMG141104	Adult, female	Shangsi, Guangxi, China
12	O. (B.) trankieni	NHMG141107	Adult, male	Shangsi, Guangxi, China
13	O. (B.) trankieni	NHMG141111	Adult, female	Shangsi, Guangxi, China
14	O. (B.) trankieni	NHMG141112	Adult, male	Shangsi, Guangxi, China
15	O. (B.) trankieni	NHMG141113	Adult, male	Shangsi, Guangxi, China
16	O. (B.) trankieni	NHMG141116	Adult, male	Shangsi, Guangxi, China
17	O. (B.) trankieni	NNU20042908	Adult, male	Wuming, Guangxi, China
18	O. (B.) trankieni	NNU20042909	Adult, male	Wuming, Guangxi, China
19	O. (B.) trankieni	NNU20042910	Adult, male	Wuming, Guangxi, China
20	O. (B.) trankieni	NNU20210304	Adult, male	Wuming, Guangxi, China
21	O. (B.) trankieni	NNU202103001	Adult, female	Wuming, Guangxi, China
22	O. (B.) trankieni	NNU202103002	Adult, female	Wuming, Guangxi, China
23	O. (B.) trankieni	NNU202103003	Adult, female	Wuming, Guangxi, China
24	O. (B.) versabilis	NNU00637	Adult, female	Jinxiu, Guangxi, China
25	O. (B.) versabilis	NNU00638	Adult, male	Jinxiu, Guangxi, China
26	O. (B.) versabilis	NNU00639	Adult, female	Jinxiu, Guangxi, China
27	O. (B.) versabilis	NNU00640	Adult, female	Jinxiu, Guangxi, China
28	O. (B.) versabilis	NNU00641	Adult, male	Jinxiu, Guangxi, China
29	O. (B.) versabilis	NNU00642	Adult, female	Jinxiu, Guangxi, China
30	O. (B.) versabilis	NNU00643	Adult, female	Jinxiu, Guangxi, China
31	O. (B.) versabilis	NNU00644	Adult, female	Jinxiu, Guangxi, China
32	O. (B.) versabilis	NNU00645	Adult, male	Jinxiu, Guangxi, China
33	O. (B.) versabilis	NNU00647	Adult, female	Jinxiu, Guangxi, China
34	O. (B.) versabilis	NNU201908005	Sub-adult	Longsheng, Guangxi, China
35	O. (B.) versabilis	NNU201908010	Adult, female	Longsheng, Guangxi, China
36	O. (B.) yentuensis	NHMG1401035	Adult, male	Shangsi, Guangxi, China
37	O. (B.) yentuensis	NHMG1401036	Adult, male	Shangsi, Guangxi, China
38	O. (B.) yentuensis	NNU00230	Adult, male	Shangsi, Guangxi, China
39	O. (B.) yentuensis	NHMG1505001	Adult, male	Shangsi, Guangxi, China
40	O. (B.) yentuensis	NHMG1505002	Adult, male	Shangsi, Guangxi, China
41	O. (B.) yentuensis	NHMG1505003	Adult, male	Shangsi, Guangxi, China
42	O. (B.) yentuensis	NHMG1505004	Adult, male	Shangsi, Guangxi, China
43	O. (B.) yentuensis	NHMG1505005	Adult, male	Shangsi, Guangxi, China
44	O. (B.) yentuensis	NHMG1505006	Adult, male	Shangsi, Guangxi, China
45	O. (B.) yentuensis	NHMG1505007	Adult, male	Shangsi, Guangxi, China
46	O. (B.) yentuensis	NHMG1505008	Adult, male	Shangsi, Guangxi, China
47	O. (B.) yentuensis	NHMG1505009	Adult, male	Shangsi, Guangxi, China
48	O. (B.) yentuensis	NHMG1505010	Adult, male	Shangsi, Guangxi, China
49	O. (B.) yentuensis	NHMG1505011	Adult, female	Shangsi, Guangxi, China
50	O. (B.) yentuensis	NHMG1505012	Adult, female	Shangsi, Guangxi, China
51	O. (B.) yentuensis	NHMG1505013	Adult, female	Shangsi, Guangxi, China
52	O. (B.) yentuensis	NHMG1505014	Adult, female	Shangsi, Guangxi, China
53	O. (B.) yentuensis	NHMG1505015	Adult, female	Shangsi, Guangxi, China
54	O. (B.) yentuensis	NHMG1505016	Adult, female	Shangsi, Guangxi, China

## Supplementary materials

Supplementary material 1 Supplementary information Data type xls Explanation note **table S1.** Uncorrected p-distance based on 16S gene fragment. **table S2.** Uncorrected *p*-distance based on COI gene fragment. https://binary.pensoft.net/file/973873This dataset is made available under the Open Database License (http://opendatacommons.org/licenses/odbl/1.0/). The Open Database License (ODbL) is a license agreement intended to allow users to freely share, modify, and use this Dataset while maintaining this same freedom for others, provided that the original source and author(s) are credited.Wei-Cai Chen, Yun-Ming Mo, Li Lin, Kun Qin

## References

[B1] Altschul SF Madden TL Schäffer AA Zhang J Zhang Z Miller W Lipman DJ (1997) Gapped BLAST and PSI-BLAST: A new generation of protein database search programs. Nucleic Acids Research 25(17): 3389–3402. 10.1093/nar/25.17.3389 9254694 PMC146917

[B2] AmphibiaChina (2023) The database of Chinese amphibians. Kunming Institute of Zoology (CAS), Kunming, Yunnan, China. http://www.amphibiachina.org/

[B3] Boulenger GA (1903) Descriptions of three new batrachians from Tonkin. Annals & Magazine of Natural History 7(12): 186–188. 10.1080/00222930308678835

[B4] Che J Chen HM Yang JX Jin JQ Jiang K Yuan ZY Murphy RW Zhang YP (2012) Universal COI primers for DNA barcoding amphibians. Molecular Ecology Resources 12(2): 247–258. 10.1111/j.1755-0998.2011.03090.x 22145866

[B5] Chen BH (1991) The Amphibian and Reptilian Fauna of Anhui. Anhui Publishing House of Science and Technology, Hefei, 408 pp.

[B6] Chen WC (2018) *Odorrananasuta*. Guangxi forestry 2018(03): 1–33.

[B7] Chen XH Chen Z Jiang JP Qiao L Lu YQ Zhou KY Zheng GM Zhai XF Liu JX (2013) Molecular phylogeny and diversification of the genus *Odorrana* (Amphibia, Anura, Ranidae) inferred from two mitochondrial genes. Molecular Phylogenetics and Evolution 69(3): 1196–1202. 10.1016/j.ympev.2013.07.023 23911727

[B8] Fei L Ye CY Huang YZ (1990) Key to Chinese Amphibians. Publishing House for Scientific and Technological Literature, Chongqing, 364 pp.

[B9] Fei L Ye CY Li C (2001) Taxonomic studies of *Odorranaversabilis* in China II Descriptions of two new species (Amphibia: Ranidae). Acta Zootaxonomica Sinica 26: 601–607.

[B10] Fei L Ye CY Huang YZ Jiang JP Xie F (2005) An Illustrated Key to Chinese Amphibians. Sichuan Publishing House of Science and Technology, Chengdu, 340 pp.

[B11] Fei L Hu SQ Ye CY Huang YZ (2009) Fauna Sinica. Amphibia (Vol. 2). Anura. Science Press, Beijing, 957 pp.

[B12] Fei L Ye CY Jiang JP (2010) Phylogenetic systematics of Ranidae. Herpetologica Sinica 12: 1–43.

[B13] Fei L Ye CY Jiang JP (2012) Colored Atlas of Chinese Amphibian. Sichuan Publishing Group, Sichuan.

[B14] Frost DR (2023) Amphibian Species of the World: an Online Reference. Version 6.1. American Museum of Natural History, New York. [Accessed 15 July 2023] 10.5531/db.vz.0001

[B15] Guo HS Zhang JX Zhu FX (1966) Surveys of amphibians of Zhejiang Province. Chinese Journal of Zoology 8(1): 31–34.

[B16] Hu SC Zhao EM Liu CC (1973) A survey of amphibians and reptiles in Kweichow Province, including a herpetofaunal analysis. Acta Zoologica Sinica 19(2): 149–178.

[B17] Hu SC Fei L Ye CY (1978) A survey of amphibians in Fujian Province. Materials for Herpetological Research 4: 22–29.

[B18] Huang MH Jin YL Cai CM (1990) Fauna of Zhejiang, Amphibia and Reptilia. Zhejiang Science and Technology Publishing House, Hangzhou, 306 pp.

[B19] Huang Y Wang B Yan LM Wei XM Lin L Wei JC Li HJ (2020) Observations on the spatial and temporal patterns of amphibian diversity in Damingshan, Guangxi. Journal of Ecology and Rural Environment 36(8): 968–974.

[B20] Kumar S Stecher G Tamura K (2016) MEGA 7: Molecular Evolutionary Genetics Analysis Version 7.0 for Bigger Datasets. Molecular Biology and Evolution 33(7): 1870–1874. 10.1093/molbev/msw054 27004904 PMC8210823

[B21] Li C Ye CY Fei L (2001) Taxonomic studies of *Odorranaversabilis* in China I. taxonomic status of the geographic populations (Amphibia: Ranidae). Acta Zootaxonomica Sinica 26: 593–600.

[B22] Liu CC Hu SQ (1962) A herpetological report of Kwangsi. Acta Zootaxonomica Sinica 14(Supplement): 73–104.

[B23] Liu CC Hu SC Fei L (1973) On collections of amphibians from Hainan Island. Acta Zootaxonomica Sinica 19(4): 385–404.

[B24] Lu LL Lv ZT Wang J Wang YY (2016) First record and redescription of *Odorranayentuensis* from China. Chinese Journal of Wildlife 37: 390–394.

[B25] Luo T Wang SW Xiao N Wang YL Zhou J (2021) A new species of odorous frog genus *Odorrana* (Anura, Ranidae) from southern Guizhou Province, China. Asian Herpetological Research 12(4): 381–398. 10.16373/j.cnki.ahr.200122

[B26] Ma JF Zong Y Wu WX (1982) New records of frogs to Jiangxi Province. Bo Wu 1: 1–37.

[B27] Miller MA Pfeiffer W Schwartz T (2010) Creating the CIPRES Science Gateway for inference of large phylogenetic trees. Proceedings of the Gateway Computing Environments Workshop (GCE), 14 Nov. 2010, New Orleans, 8 pp. 10.1109/GCE.2010.5676129

[B28] Mo YM Wei ZY Chen WC (2014) Colored Atlas of Guangxi Amphibians. Guangxi Science and Technology Publishing House, Nanning, 313 pp.

[B29] Nylander JAA (2004) MrModeltest v 2: Program distributed by the author. Evolutionary Biology Centre, Uppsala University.

[B30] Orlov NL Ngat LN Cuc HT (2003) A new species of cascade frog from north Vietnam (Ranidae, Anura). Russian Journal of Herpetology 10(2): 123–134. 10.30906/1026-2296-2003-10-2-123-134

[B31] Pan JH Liu CH Qian XG (1985) Studies on amphibian fauna of the mainland and some adjacent islands of Guangdong Province. Acta Herptetologica Sinica 4(3): 200–208.

[B32] Pham CT Le MD Ngo HT Nguyen TQ (2020) New records of Cascade Frogs of the genus *Odorrana* (Amphibia: Anura: Ranidae) from Vietnam. Academia Journal of Biology 42(4): 33–40. 10.15625/2615-9023/v42n4.15244

[B33] Ronquist F Teslenko M Van Der Mark P Ayres DL Darling A Höhna S Larget B Liu L Suchard MA Huelsenbeck JP (2012) Mrbayes 3.2: Efficient bayesian phylogenetic inference and model choice across a large model space. Systematic Biology 61(3): 539–542. 10.1093/sysbio/sys029 22357727 PMC3329765

[B34] Sichuan Institute of Biology (1974) A herpetological survey of Anhui Province. Materials for Herpetological Research 2: 48–57.

[B35] Smith MA (1921) New or little-known reptiles and batrachians from southern Annam (Indo-China). Proceedings of the Zoological Society of London 1921(2): 423–440. 10.1111/j.1096-3642.1921.tb03271.x

[B36] Stuart BL Chan-ard T (2005) Two new *Huia* (Amphibia: Ranidae) from Laos and Thailand. Copeia 2005(2): 279–289. 10.1643/CH-04-137R3

[B37] Tran TT Orlov NL Nguyen TT (2008) A new species of Cascade Frog of *Odorrana* Fei, Yi et, Huang, 1990 genus (Amphibia: Anura: Ranidae) from Bac Giang Province (Yen Tu Mountain Range, northeast Vietnam). Russian Journal of Herpetology 15: 212–224.

[B38] Wu GF (1977) A new species of frogs from Huang-Shan, Anhui, *Ranatormotus* Wu. Acta Zoologica Sinica 23: 113–115.

[B39] Wu L Dong Q Xu RH (1986) The Amphibian Fauna of Guizhou. Guizhou People’s Press, Guiyang.

[B40] Yang DT (1991) Phylogenetic systematics of the Amolops group of ranid frogs of southeastern Asia and the Greater Sunda Islands. Fieldiana 63: 1–31. 10.5962/bhl.title.2854

[B41] Zhang YX Wen YT (2000) Guangxi Amphibians. Guangxi Normal University Press, Guilin, 183 pp.

[B42] Zong Y Ma JF (1985) A survey of amphibians and reptiles of Mt. Jinggang region in Jiangxi Province. Investigation and Studium 5: 167–171.

[B43] Zou DL (1983) Surveys of amphibians in Jiulianshan area, Jiangxi. Journal of Jiangxi University 1983(2): 5–10.

